# Die optische Kohärenztomographie-Angiographie und Erkrankungen des kardiovaskulären Spektrums. Ein Überblick über die aktuelle Studienlage

**DOI:** 10.1007/s00347-021-01336-1

**Published:** 2021-02-22

**Authors:** Martin Dominik Leclaire, Nicole Eter, Maged Alnawaiseh

**Affiliations:** 1 Klinik für Augenheilkunde, Universitätsklinikum Münster, Domagkstr. 15, 48149 Münster, Deutschland; 2 Klinik für Augenheilkunde, Klinikum Fulda, Universitätsmedizin Marburg – Campus Fulda, Fulda, Deutschland

**Keywords:** OCT‑A, Kardiovaskuläre Erkrankungen, Bildgebung, Netzhaut, Aderhaut, OCTA, Cardiovascular diseases, Imaging, Retina, Choroid

## Abstract

**Hintergrund:**

Kardiovaskuläre Erkrankungen (KVE) sind die Haupttodesursache weltweit. Die Beobachtbarkeit von Veränderungen der retinalen Gefäße im Zusammenhang mit KVE mittels Fundoskopie ist schon seit Langem bekannt. Ein neuartiges Verfahren zur nichtinvasiven und detaillierten Darstellung und Quantifizierung der retinalen und papillären Gefäße stellt die optische Kohärenztomographie-Angiographie (OCT-A) dar. Durch die OCT‑A ist es möglich, Gefäßveränderungen einfach und gut reproduzierbar zu visualisieren, weswegen ihr Einsatz nicht nur auf augenärztliche Fragestellungen beschränkt ist. In den vergangenen Jahren sind einige experimentelle und klinische Studien zur Darstellbarkeit und Quantifizierung von Gefäßveränderungen durch die OCT‑A bei Erkrankungen des kardiovaskulären Spektrums publiziert worden. In der vorliegenden Übersicht werden die wichtigsten Erkenntnisse aus diesen Studien zusammengefasst.

**Methoden:**

Grundlage dieser Arbeit bilden eine umfassende selektive Literaturrecherche und die Darstellung eigener Daten.

**Ergebnisse:**

Mittlerweile liegen zu vielen Erkrankungen des kardiovaskulären Spektrums OCT-A-Studien vor, die verdeutlichen, dass systemische Gefäßerkrankungen mit Veränderungen der retinalen Mikrozirkulation verbunden sind. Mit der OCT‑A können diese Veränderungen visualisiert und reproduzierbar quantifiziert werden. Oftmals ist es möglich, subklinische Veränderungen aufzuzeigen, bevor die zugrunde liegende Erkrankung anderweitig messbare Veränderungen oder für den Patienten merkbare Symptome verursacht.

**Schlussfolgerung:**

Die OCT‑A ist eine vielversprechende Bildgebungsmethode auf dem Gebiet der KVE in Wissenschaft und klinischer Anwendung. Sie kann zur Diagnostik und Quantifizierung retinaler Gefäßveränderungen eingesetzt werden. Weitere Studien werden zeigen, ob die OCT‑A bei der Einschätzung des individuellen kardiovaskulären Risikoprofils helfen kann.

## Abstract (en)

**Background:**

Cardiovascular diseases (CVD) are the leading cause of death worldwide. The observability of retinal vessel changes in the context of CVD by funduscopy has been known for a long time. Optical coherence tomography angiography (OCTA) is a novel technique for noninvasive detailed imaging and quantification of the retinal microcirculation. The OCTA enables the visualization of vascular changes in a simple and well-reproducible way. Its applications are not limited to the field of ophthalmology. In recent years, several experimental and clinical studies have been published on vascular changes detectable with OCTA in CVD. This review summarizes the most important findings of these studies.

**Methods:**

This work is based on a comprehensive review of the literature and on the presentation of own data.

**Results:**

Nowadays, OCTA studies are available for many diseases of the cardiovascular spectrum. This indicates that systemic vascular diseases are associated with changes in the retinal microcirculation. These changes can be visualized and reproducibly quantified with OCTA. In many diseases subclinical changes, which are detectable by OCTA, occur before the underlying disease causes otherwise measurable changes or noticeable symptoms for the patient.

**Conclusion:**

The OCTA is a promising imaging method in the context of CVD in both scientific and clinical applications. It can be used for the diagnostics and quantification of retinal vascular changes. Further studies will show whether OCTA can be of assistance in the estimation of individual systemic cardiovascular risk profiles.

## Bedeutung und Definition kardiovaskulärer Erkrankungen

Kardiovaskuläre Erkrankungen (KVE) sind heute bereits weltweit die Haupttodesursache [71] und führen zu enormen volkswirtschaftlichen Kosten. Veränderte Lebensgewohnheiten sowie eine alternde Bevölkerung lassen die Prävalenz der KVE weltweit und insbesondere in Entwicklungsländern steigen [44]. Die Definition der KVE ist nicht eindeutig. So versteht die WHO den Begriff in weiterem Sinne und zählt die koronare Herzerkrankung (KHK), die periphere arterielle Verschlusskrankheit (pAVK), zerebrovaskuläre Erkrankungen, die tiefe Beinvenenthrombose und Lungenembolie, aber auch kongenitale Herzerkrankungen und rheumatisches Fieber dazu [78]. Als wichtigste Risikofaktoren nennt die Weltgesundheitsorganisation (WHO) u. a. die arterielle Hypertonie, Tabakrauchen, körperliche Inaktivität, Diabetes mellitus und Hyperlipidämie.

## Die Quantifizierung retinaler Gefäßveränderungen und die optische Kohärenztomographie-Angiographie

Durch die klaren optischen Medien sind die Netzhautgefäße mittels Fundoskopie einfach zu beobachten. Dieser Umstand ist seit Langem bekannt [27], und in der Vergangenheit wurden verschiedene Klassifizierungen zu fundoskopisch sichtbaren Gefäßveränderungen etabliert, etwa nach Keith-Wagener-Barker [35] zur hypertensiven Retinopathie oder die modifizierte Arlie-House-Klassifizierung zur diabetischen Retinopathie [77]. Letztere basiert auf stereoskopischen Fundusfotos. Fundusfotos wurden über Jahrzehnte in der augenheilkundlichen Forschung und in der klinischen Praxis angewandt. Die Analyse von Gefäßveränderungen auf Fundusfotos konzentrierte sich u. a. auf den Parameter Gefäßweite, auf das Verhältnis von Arteriolen- zu Venolendurchmesser („arteriovenous ratio“), an dem Winkel an den Gefäßverzweigungen, an dem Verhältnis von Gefäßlänge zu Durchmesser („length:diameter ratio“) und an dem Ausmaß einer Gefäßtortuositas [57]. Für eine detaillierte Übersicht über die Möglichkeiten der Bildanalyse von Fundusfotografien sei auf die Arbeit von Patton et al. verwiesen [57]. Weiterhin sind verschiedene Analysemethoden der okulären Durchblutung wie die Farbduplexsonographie, die Laser-Doppler-Velocimetrie oder die Laser-Doppler-Flussmessung beschrieben. Diese Methoden wurden genutzt, um genauere Informationen über die retinale Durchblutung zu gewinnen [58, 61]. Der Einsatz dieser Methoden hat sich jedoch aufgrund von verschiedenen Limitationen nicht durchgesetzt.

**Figure 1:**
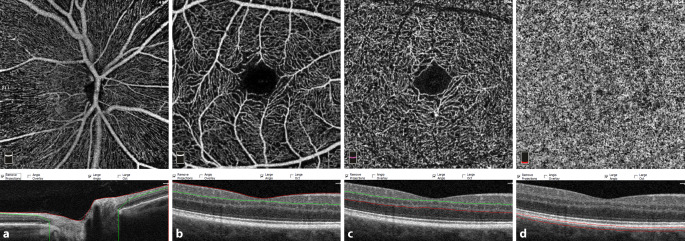

Die optische Kohärenztomographie-Angiographie (OCT-A) basiert auf der Technologie der optischen Kohärenztomographie (OCT) und ist in der Lage, durch mehrfaches, hochauflösendes Scannen eines bestimmten Netzhautbereiches die durch die Bewegung der Blutzellen verursachten Signalunterschiede zu erfassen und dadurch Blutgefäße zu visualisieren. Die OCT‑A ermöglicht die detaillierte Darstellung kleiner Gefäße und Kapillaren in den unterschiedlichen Netzhautschichten und in der Choriokapillaris (beispielhaft dargestellt in Abb. 1).

Im Vergleich zur Fundusfotografie ist die OCT‑A aufgrund der hohen Auflösung und der standardisierten Untersuchungstechnik in der Lage, die Gefäßarchitektur bis hin zu den kleinen Kapillaren darzustellen und automatisiert einfach und schnell zu quantifizieren. Im Gegensatz zur Fundoskopie bzw. der Auswertung von Fundusfotos erzeugt die OCT‑A dreidimensionale Bilder. Dadurch ist eine segmentale Analyse in den verschiedenen Netzhautschichten und in der Choriokapillaris möglich. Ferner ist mittels OCT‑A eine Darstellung und Quantifizierung der fovealen avaskulären Zone verfügbar, ein Parameter, der bei vielen okulären und systemischen Erkrankungen verändert ist [51]. Die OCT‑A benötigt anders als herkömmliche Bildgebungsmethoden wie Fluoreszeinangiographie und Indocyaningrünangiographie keinen Farbstoff, weshalb ihre Anwendbarkeit auch bei Unverträglichkeit, schwererer Niereninsuffizienz oder Schwangerschaft gegeben ist. Ferner wird die Darstellung nicht durch Überlagerungsphänomene wie etwa bei Gefäßleckagen gestört. Durch die standardisierte Aufnahmetechnik, die vorgeschriebene Scangröße und die Anwendung von Eyetracking-Systemen ist es möglich, genau definierte Bereiche der Netzhaut reproduzierbar darzustellen. Darauf folgend wurden verschiedene Parameter in den letzten Jahren etabliert, welche die Gefäßarchitektur und die retinale Mikrozirkulation quantitativ beschreiben. Zu den wichtigsten erhobenen Parametern zählen die Gefäßdichte („vessel densitiy“ [VD]) und die Größe der fovealen avaskulären Zone (FAZ) [34]. Die Wiederholbarkeit und Reproduzierbarkeit dieser quantitativen Parameter wurden bei gesunden Probanden, bei ophthalmologischen Erkrankungen und im Tiermodell gut untersucht [3, 8, 36, 50, 75]. Auch bei verschiedenen ophthalmologischen und systemischen Erkrankungen zeigen diese Parameter Korrelationen mit der Ausprägung der Erkrankung [5–7, 13, 18, 23, 41, 42, 48, 60, 72, 74]. Die Möglichkeit der nichtinvasiven, schnellen und einfachen Gefäßdarstellung und -quantifizierung mittels OCT‑A ist einzigartig. Es ist bekannt, dass ein Zusammenhang zwischen retinalen Gefäßveränderungen und dem Risiko für eine kardiovaskulär bedingte Mortalität besteht [10]. Daher stellt die OCT‑A eine vielversprechende Bildgebungsmodalität zur präzisen und nichtinvasiven Darstellung und Quantifizierung dieser Gefäßveränderungen dar.

## Methoden

Der vorliegenden narrativen Übersichtsarbeit liegt eine umfassende selektive Literaturrecherche bei PubMed [79] zugrunde. Hierzu wurden die jeweilige Erkrankung sowie die Begriffe „Optical coherence tomography angiography“ als Suchbegriffe verwendet. Ferner wurden die Daten aus Arbeiten aus der eigenen Arbeitsgruppe zum Thema zusammengefasst. Für die Darstellung der Ergebnisse der OCT‑A im Hinblick auf die verschiedenen Erkrankungen wurden Originalarbeiten hinzugezogen. Für die Einleitung, die Schlussfolgerung und den einleitenden Satz zu den jeweiligen Erkrankungen wurden auch Übersichtsarbeiten berücksichtigt.

## Arterielle Hypertonie – die Rolle der Netzhautgefäße

Die arterielle Hypertonie ist ein wesentlicher Risikofaktor für die Entstehung ischämischer und hämorrhagischer Schlaganfälle, Myokardinfarkte, Herzinsuffizienz und pAVK und somit eine der Hauptursachen für vorzeitige Todesfälle [59]. Die Ausprägung der hypertensiven Retinopathie gilt schon seit Langem als Indikator für das Auftreten und die Mortalität bei kardiovaskulären Ereignissen wie Schlaganfällen [49] und Myokardinfarkten [53] und damit für die Gesamtmortalität bei Patienten mit arterieller Hypertonie. Dementsprechend wurden seit der Entwicklung der OCT‑A mehrere Studien mit Hypertonuspatienten durchgeführt.

Es konnte gezeigt werden, dass VD und Gefäßlänge („vessel lengths“ [VL]) gemessen mittels OCT‑A in der fovealen Choriokapillaris negativ mit dem Grad einer hypertensiven Retinopathie (nach Keith-Wagener-Baker) korreliert sind (*n* = 361 gesunde Probanden und *n* = 206 Patienten mit arteriellem Hypertonus, *p* < 0,001 für VD und VL), wobei die Messung der Gefäßparameter in der fovealen Choriokapillaris im Gegensatz zur makulären Choriokapillaris stark mit der Ausprägung der hypertensiven Retinopathie, aber nicht mit anderen Faktoren wie Alter, Geschlecht oder dem systemischen Blutdruck korreliert war [65]. Auch die Krankheitsdauer scheint einen Einfluss auf die retinalen Gefäße zu haben: Patienten mit einer länger als 5 Jahre bestehenden arteriellen Hypertonie (*n* = 52) wiesen in einer Studie von Lim et al. eine signifikant verringerte VD und Flussdichte verglichen mit Patienten mit einer Krankheitsdauer von unter 5 Jahren (*n* = 32) und verglichen mit gesunden Kontrollen (*n* = 117) auf (*p* < 0,05 in den Bereichen 1 mm und 3 mm perifoveal). Darüber hinaus war die Größe der FAZ bei den Patienten mit länger bestehender arterieller Hypertonie (>5 Jahre) signifikant erhöht im Vergleich zu gesunden Probanden (*p* = 0,019) und zu Patienten mit kürzer bestehender arterieller Hypertonie (<5 Jahre; *p* = 0,004). Bemerkenswerterweise war das Patientenkollektiv in dieser Arbeit jung (28,3 ± 2,8 Jahre) und der Blutdruck medikamentös gut eingestellt. In Zusammenschau mit einer ebenfalls festgestellten Ausdünnung der inneren plexiformen Schicht wurde eine retinale Ischämie durch eine Störung der retinalen Mikrozirkulation durch eine länger bestehende arterielle Hypertonie postuliert [43]. In einer anderen Fall-Kontroll-Studie wiesen Sun et al. bei Patienten mit arterieller Hypertonie (*n* = 94) im Vergleich mit gesunden Kontrollen (*n* = 46) eine signifikant verringerte VD im tiefen makulären Gefäßplexus (*p* = 0,002) sowie ebenfalls eine vergrößerte FAZ (*p* = 0,003) nach [64]. Die Ergebnisse konnten von einer weiteren Studie mit 30 Patienten mit arterieller Hypertonie und 30 gesunden Kontrollen bestätigt werden: Hier wurden ebenfalls eine signifikant verringerte VD im tiefen Gefäßplexus makulär und eine Vergrößerung der FAZ beobachtet (*p* < 0,05) [24].

Diese Ergebnisse sind auch im Hinblick auf eine arterielle Hypertonie als möglichen Bias in der Auswertung von OCT-A-Daten für wissenschaftliche Zwecke von Bedeutung.

Es konnte im Vergleich zu alterskorrelierten gesunden Probanden gezeigt werden, dass Patienten mit einer kürzlich stattgefundenen (≤7 Tage) hypertensiven Krise (Blutdruckwerte systolisch ≥ 180 mm Hg und/oder ≥ 110 mm Hg diastolisch) eine signifikant reduzierte VD und „skeleton density“ (ein Derivat der Gefäßdichte) im tiefen makulären Plexus aufweisen (*p* = 0,004 bzw. 0,002, *n* = 28 Augen von 17 Patienten und *n* = 31 Augen von 18 Kontrollprobanden). Ferner wurden signifikante Veränderungen in der choroidalen Gefäßarchitektur, u. a. charakterisiert durch signifikant vermehrte und vergrößerte Leerräume zwischen den Flusssignalen („voids“), nachgewiesen (*p* < 0,05) [68].

## Herzinsuffizienz

Die chronische Herzinsuffizienz ist charakterisiert durch eine strukturelle oder funktionelle Verringerung der Ventrikelfüllung oder der Blutejektion, und ihre Prävalenz steigt weltweit an [76]. Mittels OCT‑A konnte gezeigt werden, dass Patienten mit chronischer Herzinsuffizienz im Vergleich zu gesunden Probanden (jeweils *n* = 25) eine signifikant verringerte VD in der Choriokapillaris aufwiesen (*p* > 0,01) [31].

Auch kongenitale Herzfehler können zur Herzinsuffizienz führen: Bei etwa 25 % der Patienten mit angeborenen Herzfehlern liegt im Alter von 30 Jahren eine Herzinsuffizienz vor [56]. In einer OCT-A-Studie wurde nachgewiesen, dass Patienten mit zyanotischen angeborenen Herzfehlern (Sauerstoffsättigung < 90 %, *n* = 57) im Vergleich mit gesunden Kontrollprobanden (*n* = 41) eine signifikant verringerte VD im oberflächlichen und tiefen makulären sowie im peripapillären Gefäßplexus („radial peripapillary capillary [RPC] plexus“) aufweisen (*p* < 0,01). Ferner waren die VD im tiefen makulären und im RPC-Plexus signifikant verringert im Vergleich zu Patienten mit azyanotischem angeborenem Herzfehler (*n* = 60, *p* < 0,05) [41].

## Koronare Herzerkrankung, akutes Koronarsyndrom und Myokardinfarkt

Die KHK zählt zu den Haupttodesursachen weltweit und ist im Wesentlichen durch eine fortschreitende Atherosklerose der Koronararterien gekennzeichnet [47]. Der Zusammenhang zwischen fundoskopisch unmittelbar sichtbaren Gefäßveränderungen der Netzhaut und dem Schweregrad einer KHK ist bereits lange vor der Ära der OCT‑A beschrieben worden [49, 67]. Wang et al. konnten in einer relativ großen prospektiven Studie bei 158 KHK-Patienten [73] (diagnostiziert mittels Koronarangiographie) im Vergleich mit gesunden Kontrollen eine signifikant verringerte VD im oberflächlichen und tiefen Gefäßplexus (mit Ausnahme der Fovea) als auch in der Choroidea in der OCT‑A nachweisen (*p* < 0,001 für alle Werte). Interessanterweise ergab sich für die Gefäße der äußeren Netzhaut eine signifikant erhöhte VD (*p* < 0,001). Im Hinblick auf die Netzhautdicke konnten keine signifikanten Unterschiede zwischen der Kontrollgruppe und den KHK-Patienten festgestellt werden. Um den Effekt der Koronararterienstenose weiter zu untersuchen, wurde die Korrelation zwischen der VD und dem Gensini-Score untersucht. Der Gensini-Score ist ein etablierter Gewichtungsfaktor für die Lokalisation der Koronararterienstenose entsprechend der Wichtigkeit der einzelnen Abschnitte der Koronararterien für die kardiale Durchblutung [26]. Die Korrelationsanalysen in dieser Arbeit entsprechend der Wichtung des Gensini-Scores ergaben, dass eine Stenose im linken Hauptstamm die VD in den meisten untersuchten retinalen und choroidalen Regionen negativ beeinflusste, gefolgt vom Ramus interventricularis anterior und vom Ramus circumflexus. Außerdem konnte gezeigt werden, dass Koronarstenosen unterschiedlicher Lokalisation die VD in unterschiedlichen Plexus in der OCT‑A beeinflussen. Bei keinem der eingeschlossenen Patienten lagen fundoskopisch sichtbare Gefäßveränderungen vor, sodass davon ausgegangen werden kann, dass die retinale Mikroarchitektur bei Vorliegen einer KHK bereits signifikante Veränderungen aufweist, bevor diese für den Augenarzt unmittelbar sichtbar werden.

Das akute Koronarsyndrom ist ein Überbegriff für akute Beschwerden ausgelöst durch eine Insuffizienz der Koronararterien. Es resultiert in der Regel aus einer Plaqueruptur in den Koronararterien mit darauf folgender Aktivierung der Gerinnungskaskade und Thrombusbildung, wodurch es zu einem partiellen oder kompletten Verschluss des betroffenen Koronargefäßes kommt [46].

Arnould et al. untersuchten in einer prospektiven Studie die VD bei Patienten mit akutem Koronarsyndrom (ACS) innerhalb von 2 Tagen nach Krankenhauseinweisung. Verglichen mit gesunden, alters- und geschlechtskorrelierten Kontrollen hatten die ACS-Patienten eine signifikant verringerte VD (*n* = 44, *p* > 0,001). Innerhalb des ACS-Patientenkollektivs (*n* = 237) zeigte sich bei den Patienten mit einer VD im unteren Drittel bezogen auf das Gesamtkollektiv ein höheres kardiovaskuläres Risikoprofil, ausgedrückt durch ein höheres Lebensalter, häufigeres Vorkommen von arterieller Hypertonie, Diabetes und pAVK sowie durch eine niedrigere linksventrikuläre Ejektionsfraktion (LVEF). Auch verschiedene Laborparameter, die mit einem erhöhten kardiovaskulären Risiko verbunden sind (Blutglukose, HbA_1c_, pro-BNP, Kreatinin und Troponin), waren in dieser Patientengruppe signifikant erhöht. Die VD korrelierte ferner signifikant mit dem Risikoprofil der Patienten für kardiovaskuläre Ereignisse (berechnet nach dem Risikoscore der American Heart Association) [11].

In einer weiteren prospektiven Studie der gleichen Arbeitsgruppe wurde untersucht, inwiefern die veränderte Hämodynamik durch einen Myokardinfarkt einen Einfluss auf die retinale VD hat. Es ergab sich keine signifikante Korrelation zwischen der LVEF, dem Herzindex (einem Indikator für die Herzleistung berechnet aus Herzminutenvolumen in Bezug zur Körperoberfläche) und der retinalen VD (*n* = 30). Dies galt sowohl für die Akutphase als auch für eine Verlaufskontrolle nach 3 Monaten [12].

## Zerebrovaskuläre Erkrankungen

Zu den zerebrovaskulären Erkrankungen („cerebrovascular disease“ [CVD]) zählen nach Definition der *American Association of Neurological Surgeons* u. a. der ischämische und der hämorrhagische Schlaganfall und die Karotisstenose [80].

Die „cerebral autosomal dominant arteriopathy with subcortical infarcts and leukoencephalopathy“ (CADASIL) ist eine durch eine Mutation im *Notch‑3*-Gen hervorgerufene Kleingefäßerkrankung und die häufigste hereditäre Ursache für ischämische Schlaganfälle. Eine Studie von Nelis et al. ergab eine hochsignifikant reduzierte VD im tiefen makulären Gefäßplexus bei CADASIL-Patienten im Vergleich mit gesunden Kontrollprobanden (jeweils *n* = 21 Augen von 11 Individuen, *p* < 0,0001) [54].

Die Karotisstenose ist ein wesentlicher Risikofaktor für ischämische Schlaganfälle und andere thrombembolische Ereignisse wie etwa retinale Arterienverschlüsse. Chirurgische Rekanalisierungsmöglichkeiten sind v. a. die Karotisendarteriektomie und das weniger invasive Stenting [21]. Lahme et al. wiesen in einer prospektiven Studie nach, dass Patienten mit schwerer asymptomatischer Karotisstenose (*n* = 25) sowohl im makulären (*p* = 0,003) als auch im papillären (*p* = 0,013) OCT-Angiogramm eine signifikant verringerte VD aufweisen als gesunde Kontrollen, wobei der Unterschied in der makulären OCT‑A auf den oberflächlichen Plexus beschränkt war. Nach Karotisendarteriektomie (*n* = 18) war die VD im peripapillären Gefäßplexus signifikant erhöht im Vergleich zu präoperativ. Interessanterweise traf dies sowohl für das ipsilateral, also auf der Seite, auf der die Endarteriektomie durchgeführt wurde, gelegene als auch für das kontralateral gelegene Auge zu (ipsilateral und kontralateral: *p* = 0,004) [37].

Vorhofflimmern (VHF) ist die häufigste kardiale Arrhythmie und ist über die Begünstigung kardiozerebraler Emboli für 25–30 % aller ischämischen Schlaganfälle verantwortlich [22]. Lang et al. untersuchten in einer Fall-Kontroll-Studie Patienten mit persistierendem und mit intermittierendem VHF (*n* = 44). Es konnte eine signifikant verringerte VD verglichen mit gesunden alterskorrelierten Kontrollen aufgezeigt werden. Dies betraf den oberflächlichen Gefäßplexus im makulären Whole-en-face-Angiogramm (*p* = 0,012) sowie dort auch die perifoveale Region (*p* = 0,016) und darüber hinaus das papilläre OCT-Angiogramm („whole-en-face“ *p* > 0,001, peripapillär *p* = 0,027). Ferner ergab sich eine signifikant verringerte VD im RPC-Plexus bei Patienten mit persistierendem Vorhofflimmern (*n* = 14) verglichen mit Patienten mit intermittierendem VHF, die zum Zeitpunkt der Untersuchung im Sinusrhythmus waren (*n* = 30). Die Autoren stellten die Hypothese auf, dass die reduzierte VD der Netzhautgefäße durch Mikroembolien, die zu keinen bemerkbaren funktionellen Einbußen geführt haben, oder durch eine reduzierte Auswurfleistung des Herzens und einer dadurch folgenden reduzierten Organdurchblutung bedingt sein könnte [38].

## Weitere kardiovaskuläre Erkrankungen

Trotz der insbesondere in den letzten Jahren stark ansteigenden Anzahl an Publikationen zu OCT-angiographisch erhobenen Parametern bei KVE ist die Studienlage nicht vollständig. So fehlen bislang (Stand 15.12.2020) etwa Untersuchungen zu Aortenaneurysmata. Diese stellen ebenfalls eine steigende Belastung der Gesundheitssysteme dar, zeigen eine steigende Prävalenz und sind assoziiert mit arterieller Hypertonie, Atherosklerose und Rauchen [2]. Analog zu den aufgeführten Arbeiten zum Einfluss des ACS und des Myokardinfarktes auf die retinale Gefäßarchitektur wären Untersuchungen bei Schlaganfallpatienten (in der Akutphase und im Langzeitverlauf) wünschenswert. OCT-A-Studien zu rheumatischem Fieber, Myokarditis und Endokarditis, die je nach Definition im weiteren Sinne auch zu den KVE zählen [33], liegen ebenfalls bis dato nicht vor.

## Einfluss von Risikofaktoren für kardiovaskuläre Erkrankungen auf die OCT-A-Parameter

Körperliche Inaktivität in Verbindung mit sitzender Tätigkeit ist einer der Hauptrisikofaktoren für die Entstehung von KVE und mit erhöhter Mortalität assoziiert [15, 40]. Der Einfluss körperlicher Aktivität auf die Messparameter in der OCT‑A ist daher Gegenstand mehrerer Studien gewesen, in denen einerseits kurzfristige Effekte auf die retinale Durchblutung vor und nach Aktivität und andererseits Unterschiede zwischen Probanden mit unterschiedlicher körperlicher Fitness untersucht wurden.

Es konnte gezeigt werden, dass nach körperlicher Aktivität die makuläre und peripapilläre VD signifikant absinkt (*n* = 13, makulär: *p* < 0,001, peripapillär: *p* = 0,007), allerdings ohne Einfluss auf die FAZ [45]. Diese Aussage konnte in einer weiteren Studie von Vo Kim et al. für den oberflächlichen Gefäßplexus bestätigt werden, im tiefen Gefäßplexus ergab sich allerdings eine zunehmende VD nach körperlicher Aktivität (*n* = 32, *p* > 0,001). Es konnte eine Korrelation zwischen den VD-Veränderungen und dem Blutdruck während des Trainings aufgezeigt werden (*p* < 0,001) [70]. Neben kurzfristigen Veränderungen in den OCT-A-Parametern wurde auch der Einfluss längerfristigen körperlichen Trainings untersucht. Schmitz et al. wiesen nach, dass bei gesunden Probanden (*n* = 58) nach 4‑wöchigem intensivem Intervalltraining die quantitativen OCT-A-Parameter signifikant verändert sind: Es zeigten sich eine signifikant verkleinerte FAZ (*p* < 0,001) und eine signifikant verringerte VD im oberflächlichen makulären Gefäßplexus (*p* = 0,004), während die peripapilläre VD signifikant zugenommen hatte (*p* < 0,001) [62]. Alten et al. wiesen eine inverse Korrelation zwischen individueller Fitness und FAZ-Größe nach und zeigten eine signifikante Abnahme der FAZ nach 4‑wöchigem hochintensivem Intervalltraining (*n* = 65) [9]*.* Eine negative Korrelation zwischen körperlicher Fitness und FAZ-Größe wurde auch von Nelis et al. herausgestellt [55].

Darüber hinaus wurden weitere Veränderungen der OCT-angiographisch erfassbaren Veränderung der Gefäßarchitektur im Hinblick auf kardiovaskulär bedeutsame Risikofaktoren beschrieben. Hierzu zählen der Diabetes mellitus, Rauchen und Adipositas.

Bei Typ-I-Diabetikern ohne Zeichen einer diabetischen Retinopathie (DR) (*n* = 25) konnte im Vergleich zu gesunden Kontrollen eine Verringerung der VD im tiefen makulären Kapillarplexus nachgewiesen werden (*p* = 0,005) [17]. Diese Beobachtung konnte in einer weiteren Studie bestätigt werden, in der jedoch zusätzlich eine signifikant verringerte VD in der para- und perifovealen Region des oberflächlichen Plexus gezeigt werden konnte (*n* = 58, *p* > 0,01) [63].

Bei Patienten mit manifester DR konnten u. a. signifikante Korrelationen zwischen dem Schweregrad der DR und der VD, der FAZ-Größe, der FAZ-Zirkularität aufgezeigt werden (*n* = 434 Augen von 286 Patienten) [66]. In einer weiteren Studie waren systemische Risikofaktoren des Diabetes mellitus wie Hyperlipidämie, Rauchen und eine eingeschränkte Nierenfunktion mit einem signifikant reduzierten Kapillardichteindex („capillary density index“ [CDI]) verbunden. Ferner war der Schweregrad der DR mit einem signifikant reduzierten CDI assoziiert (*n* = 100 Augen von 50 Patienten) [69].

Hinsichtlich des Einflusses des Zigarettenrauchens und des Nikotins auf die OCT-A-Parameter gibt es unterschiedliche Ergebnisse. Ciesielski et al. konnten in einer Studie (*n* = 30 Augen von 30 Probanden) keine signifikanten akuten Veränderungen in den OCT-A-Parametern der Makula und des Sehnervenkopfes direkt und 30 min nach dem Rauchen einer Zigarette aufzeigen [19], auch in einer Studie von Holló ergaben sich keine signifikanten Veränderungen der peripapillären und makulären VD unmittelbar nach dem Rauchen (*n* = 7 Augen von 7 Probanden) [30]. In einer anderen Studie zeigte sich allerdings eine signifikante Abnahme der choriokapillären Durchblutung 5, 30 und 90 min nach Rauchen (*p* < 0,001). Im Vergleich zu alters- und geschlechtskorrelierten Nichtrauchern konnte die Studie allerdings keine signifikant veränderten OCT-A-Parameter bei Rauchern aufzeigen (*n* = 40 Raucher und *n* = 40 Nichtraucher) [14]. Außer dem Zigarettenrauchen wurde auch der Einfluss von Nikotinaufnahme durch Nikotinkaugummis untersucht. Hierbei zeigte sich nach 1 h Kauen (Nikotinkaugummi mit 4 mg Nikotin, *n* = 18) eine signifikant reduzierte makuläre VD (*p* < 0,05) im Vergleich zu Probanden mit Placebokaugummi (*n* = 18) [20].

Ein erhöhter BMI scheint mit veränderten OCT-A-Parametern verbunden zu sein. In einer Fall-Kontroll-Studie zwischen nichtadipösen und adipösen Frauen (*n* = 30 adipöse und *n* = 31 nichtadipöse Probandinnen, adipös definiert als Body-Mass-Index > 30 kg/m^2^) ergaben sich signifikant reduzierte VD im oberflächlichen und im tiefen makulären Gefäßplexus [32].

In einer prospektiven Studie wurden bei adipösen Patienten (*n* = 60) eine signifikant verringerte FAZ (*p* < 0,01) und ein signifikant höherer CDI im oberflächlichen Plexus (*p* = 0,04) und im tiefen Plexus (*p* = 0,01) gemessen. Es ergab sich kein Unterschied in den OCT-A-Parametern zwischen der Erstuntersuchung und dem 3‑Monats-Follow-up nach durchgeführter bariatrischer Chirurgie (*n* = 30) trotz deutlicher Gewichtsreduzierung [1].

## Zusammenfassung

Durch die Entwicklung der OCT‑A steht eine neue, nichtinvasive und schnell durchführbare Methode zur Verfügung, um die retinalen Gefäße zu beurteilen. Die Anzahl an Publikationen bezüglich der OCT‑A und systemischen Erkrankungen im Allgemeinen und bezüglich KVE im Speziellen nimmt stetig zu. Dies ist vor dem Hintergrund der steigenden Prävalenz dieser Erkrankungen zu begrüßen. Die dargestellten Ergebnisse unterstreichen, dass die OCT‑A auch außerhalb der Augenheilkunde eine vielversprechende Bildgebungsmodalität für klinische und wissenschaftliche Fragestellungen ist.

Insgesamt legen die Ergebnisse einerseits nahe, dass im Akutstadium kardiovaskulärer Ereignisse, wie etwa bei akutem Koronarsyndrom [11], Veränderung der retinalen Gefäße auftreten. Dies ist besonders interessant für die Anwendung der OCT‑A etwa in der Notfall- und Intensivmedizin. Hessler et al. verweisen darauf, dass die OCT-A-Technologie prinzipiell nicht auf den Einsatz am Auge beschränkt bleiben muss, sondern in Zukunft auch ein Einsatz an Haut oder Schleimhaut (analog zur Videomikroskopie etwa sublingual) denkbar wäre [29].

Andererseits weisen die Ergebnisse der bisherigen Studien darauf hin, dass Veränderungen der Mikrozirkulation auch vor Eintreten eines akuten kardiovaskulären Ereignisses nachweisbar sind, etwa bei Vorliegen von Risikofaktoren wie Diabetes mellitus [17, 63] und Adipositas [1, 32] und bei Erkrankungen wie asymptomatischem Vorhofflimmern [38] oder asymptomatischer Karotisstenose [37]. Bei einigen Erkrankungen sind die Veränderungen der OCT-A-Parameter auch dann nachweisbar, wenn fundoskopisch keine retinalen Gefäßveränderungen vorliegen [17, 43], sodass die OCT‑A die Möglichkeit bietet, auch subklinische Veränderungen der Gefäßarchitektur nachzuweisen, was in Zukunft zu prognostischen Zwecken genutzt werden könnte.

Die Möglichkeit zur Beurteilung der Mikrozirkulation mittels OCT‑A erfährt auch bei den Kollegen anderer Fachdisziplinen großes Interesse, was etwa im Gespräch mit Kollegen zur Planung von Studien auffällt und durch die zunehmende Anzahl an Publikationen und Arbeitsgruppen, die sich mit dem Thema auch außerhalb der Augenheilkunde beschäftigen [4, 29], bestätigt wird. Das liegt zum einen an den Vorteilen der OCT‑A als einer schnell reproduzierbaren und nichtinvasiven Untersuchung. Zum anderen liegt dies an der der Tatsache, dass die Beurteilung der Mikrozirkulation bei verschiedenen Erkrankungen immer noch eine große Problematik in der klinischen Routine darstellt und es hierzu keine etablierten Methoden für den klinischen Alltag gibt [29]. Auch wenn die OCT‑A aufgrund der erwähnten Vorteile für diese Anwendung vielversprechend ist, müssen große Herausforderungen vor der Etablierung in der klinischen Routine überwunden werden. Dies gilt insbesondere in Bezug auf Artefakte [25, 28, 39, 52] und die Identifizierung und Berücksichtigung von mehr oder weniger relevanten Einflussfaktoren wie Alter, Tagesschwankungen oder verschiedene ophthalmologische Erkrankungen [16]. Daher besteht hier ein großer Bedarf insbesondere an Langzeitstudien, die vorzugsweise therapeutische Optionen mit berücksichtigen und die OCT‑A mit etablierten Methoden vergleichen, um die Möglichkeit zum Einsatz in der klinischen Routine besser einordnen zu können und die Frage beantworten zu können, ob in Zukunft etwa Screeninguntersuchungen von Patienten mit kardiovaskulären Erkrankungen mittels OCT‑A sinnvoll wären.
